# Physician experiences with teleconsultations amidst conflict in Sudan

**DOI:** 10.1038/s41598-023-49967-5

**Published:** 2023-12-20

**Authors:** Mohammed Mahmmoud Fadelallah Eljack, Yasir Ahmed Mohammed Elhadi, Esra Abdallah Abdalwahed Mahgoub, Khabab Abbasher Hussien Mohamed Ahmed, Malaz Tarig Abd Alla Mohamed, Walaa Elnaiem, Asma Mohamedsharif, Alshareef B. Nour, Abubakr Elsadig Musa Muhammed, Mohammed Salah M. Gebril, Muhammad Sohaib Asghar, Austen El-Osta

**Affiliations:** 1 Faculty of Medicine and Health Sciences, University of Bakht Alruda, Ad Duwaym, Sudan; 2 Global Health Focus, Khartoum, Sudan; 3 Community Department, Faculty of Medicine, University of Alneelain, Khartoum, Sudan; 4 Faculty of Medicine, University of Khartoum, Khartoum, Sudan; 5 Faculty of Health Sciences and Medicine, University of Lucerne, Lucerne, Switzerland; 6 Wad Medani College of Medical Sciences and Technology, Wad Medani, Sudan; 7 Faculty of Medicine, University of Gezira, Wad Medani, Sudan; 8 Division of Nephrology and Hypertension, Mayo Clinic, Rochester, MN USA; 9 Department of Primary Care and Public Health, School of Public Health, Imperial College London, London, UK

**Keywords:** Health care, Health services

## Abstract

The current conflict in Sudan severely hinders the accessibility of health services across the country. To address this, several initiatives were proposed including offering services using teleconsultations. This study aimed to assess Sudanese doctors' teleconsultation experience, perception, and concerns during the recent conflict. This cross-sectional survey focused on Sudanese medical officers, residents, specialists, and consultants living inside or outside the country having a practice license from the Sudan Medical Council and conducting teleconsultations with Sudanese patients during the conflict period. The questionnaire was distributed to personal and professional contacts and via social media platforms in the English language among doctors who provided teleconsultation during the conflict. Data analysis was performed using the Statistical Package for Social Sciences software version 26. The study enrolled 2463 clinicians from 17 different specialties, and females represented more than half the sample (56.8%). Internal medicine was the most frequent specialty (36.1%) and the majority (68.7%) of clinicians had less than 5 years of work experience. Voice call was the most frequent platform (50.1%) used for teleconsultation during the conflict and had the highest convenience score (p < 0.01), whereas messaging platforms had the lowest score. Most clinicians (73.3%) agreed that teleconsultations created a trusted patient-physician relationship and provided good-quality care (61.8%). However, 85.1% highlighted the importance of physical touch in medical practice. Clinicians were concerned that incomplete information (81.4%), missed diagnosis (76.8%), medicolegal problems (71.0%), and prescription errors (68.4%) could arise with teleconsultations. Most respondents (70.7%) emphasized the importance of continuing to offer teleconsultation even after the war abated. In conclusion, physicians who participated in the current study agreed that teleconsultation provided quality care even in this dire crisis in Sudan. Based on our study findings, we recommend upscaling telemedicine interventions including teleconsultations at the national level. This would require unified coordination efforts of a wide mix of stakeholders to address concerns identified in the current study.

## Introduction

Recent advances in information technology coupled with the need to maintain social distancing during the COVID-19 pandemic accelerated the near-pervasive use of remote teleconsultations. This development has partially changed the traditional face-to-face communication between patients and healthcare professionals potentially transforming the provision of medical services permanently^1,2^.

Telehealth also referred to as telemedicine or e-medicine, is the remote delivery of healthcare services over the telecommunications infrastructure. Telemedicine encompasses a range of services including medical consultations and medical education as well as disease prevention, diagnosis, treatment, monitoring, and rehabilitation services^3–6^. Telehealth services are ideally provided synchronously via videoconferencing for example, or asynchronously via short messaging service (SMS) or voice records^7,8^.

The telemedicine modality is gaining a lot of momentum internationally as it enables feasible access to healthcare services and provides a satisfying experience to individuals and healthcare professionals^9–11^. Previous research has shown that telemedicine can improve the efficiency and quality of healthcare services, especially in primary healthcare settings^12^. Teleconsultation currently also has important prospects for inpatient and outpatient care, concerning modes of application of the services and the different fields of medical practice it is used in^2^. In specialties such as paediatrics, it provides an opportunity for emergency and critical care consultation, and for paediatric subspecialists to expand the reach of their patient care to distant urban and rural areas^13^. In other fields such as dermatology, neurology, and oncology patient's follow-up have also reported relative degrees of satisfaction with teleconsultations^14–16^. Despite these reported benefits, the widescale implementation of electronic health solutions in Sudan is limited to e-learning^17^ whereas the use of e-health tools in service delivery in Sudan has not been fully evaluated yet.

On 15 April 2023, a military conflict erupted in Sudan between the Rapid Support Forces (a para-military force) and the Sudan Armed Forces leading to the displacement of 2.7 million people and 444,000 refugees across the borders seeking safety^18^. Physicians who are remote providers of healthcare services were key contributors in maintaining medical services during the ongoing war in Sudan, including delivering consultations using telemedicine approaches. The delivery of telemedicine services was proposed by different national associations including the Sudanese Association of Physicians, family doctors, obstetricians, gynecologists, and paediatricians, and external groups including an alliance of Sudanese doctors in Qatar.

Despite these rapid developments, data on the application of telemedicine in Sudan remains scarce. The ongoing armed conflict in Sudan and the impact this has had on the delivery of healthcare calls for an urgent investigation to highlight the benefits of teleconsultations and to identify areas for improvement. This study aimed to assess doctors' experience, perceptions and concerns regarding the routine use of teleconsultation since the advent of the armed conflict in Sudan.

## Results

### Participant characteristics

A total of 2463 physicians completed the questionnaire. More than half of the respondents were females (56.8%, n = 1400), aged less than 30 years old (63.7%, n = 1568), had less than 5 years of working experience (68.7%, n = 1693), and spanned one of 17 different specialties. More than a third of respondents specialised in internal medicine (36.1%, n = 890), 15.1% (n = 371) were surgeons, and 13.9%, (n = 343) were paediatricians. The demographic profile of participants is shown in Table 1. Regarding the demographic of the serviced patient population, 47.3% (n = 1164) of participants provided teleconsultations for both adult and paediatric patients, whereas 41.9% (n = 1032) provided services for adults only, and 10.8% (n = 267) provided services for paediatric patients only; Table 1.

**Table 1:** Characteristics of respondents and their patient population (N = 2,463).

Variables	Categories	n	%
Sex	Male	1063	43.2
	Female	1400	56.8
Age (years)	≤ 30	1568	63.7
	31–40	640	26.0
	41–50	160	6.5
	51–60	74	3.0
	> 60	21	0.9
Years in practice	≤ 5	1693	68.7
	6–10	466	18.9
	11–20	203	8.2
	21–30	63	2.6
	> 30	38	1.5
Department	Anaesthesia	2	0.1
	Dermatology	42	1.7
	Emergency medicine	173	7.0
	ENT	33	1.3
	Family medicine	179	7.3
	Intensive care	59	2.4
	Internal medicine	890	36.1
	Obstetrics and gynaecology	200	8.1
	Oncology	23	0.9
	Ophthalmology	1	0.0
	Orthopaedics	78	3.2
	Paediatrics	343	13.9
	Physiotherapy	1	0.0
	Psychiatry	31	1.3
	Pulmonary	13	0.5
	Radiology	24	1.0
	Surgery	371	15.1
Practice location	Khartoum state	894	36.3
	Other Sudan States	1037	42.1
	Africa	77	3.1
	Asia	356	14.5
	Europe	73	3.0
	Northern and Southern America	26	1.1
Patient population	Adults	1032	41.9
	Paediatrics	267	10.8
	Both	1164	47.3

### Teleconsultation platforms and perceived convenience

The most frequent platform used for teleconsultation was phone calls (50.1%, n = 1233), followed by messaging applications (30.6%, n = 754). Video and email users showed higher appreciation for features such as ease of use, the option to have multiple participants in a call, good customer service, integration with calendars, and emergency services. On the other hand, video and telemedicine software users denoted the ability to share screens, while users of video and messaging applications highlighted that the ability to message patients is among the most important to them (Table 2). The most convenient platform for teleconsultation was video calls which had a statistically higher convenience score compared to other platforms (p < 0.001), while messaging applications had the lowest convenience score (Table 3).

**Table 2:** Teleconsultation platforms used by Sudanese doctors and their convenience (N = 2463).

N (%)		Platform convenience %							
		Response	Easy to use	Ability to have multiple people on the same call	Ability to share screen	Good customer service	Ability to message patients	Integration with calendar	Integration with emergency
Email	106 (4.3)	Disagree	1.9	9.4	6.6	1.9	5.7	3.8	5.7
		Neutral	15.1	20.8	22.6	16.0	16.0	20.8	16.0
		Agree	83.0	69.8	70.8	82.1	78.3	75.5	78.3
Messages apps	754 (30.6)	Disagree	3.8	26.3	16.4	8.6	2.3	6.5	22.7
		Neutral	17.1	19.8	22.4	30.5	15.0	28.1	24.0
		Agree	79.0	54.0	61.1	60.9	82.8	65.4	53.3
Voice call	1233 (50.1)	Disagree	4.0	29.8	19.1	8.4	8.6	5.5	15.5
		Neutral	17.5	18.1	21.1	22.3	14.5	25.5	17.1
		Agree	78.5	52.1	59.8	69.3	76.9	69.0	67.4
Telemed software	108 (4.4)	Disagree	1.9	13.9	5.6	2.8	6.5	4.6	14.8
		Neutral	15.7	24.1	15.7	15.7	16.7	22.2	21.3
		Agree	82.4	62.0	78.7	81.5	76.9	73.1	63.9
Video call	262 (10.6)	Disagree	1.5	8.4	3.8	2.3	3.4	2.7	13.4
		Neutral	12.2	9.9	8.0	14.1	9.9	13.4	17.6
		Agree	86.3	81.7	88.2	83.6	86.6	84.0	69.1

Agree = strongly agree + Agree; Disagree = strongly disagree + disagree.

**Table 3:** Convenience and concern scores of teleconsultation platforms.

	Convenience score			Concern score		
	Mean	SD	p value	Mean	SD	p value
Teleconsultation platforms			< 0.001			< 0.001
Video calls	21.7	4.2		14.3	3.0	
Email	20.6	4.0		12.7	2.6	
Telemedicine software	20.5	4.3		12.7	2.8	
Voice call	19.1	4.6		13.3	2.9	
Messages apps	18.8	4.2		13.2	2.8	

*N.B: the statistical significance may be influenced by the large size of the study groups, which can result in relatively small differences being deemed significant.

### Physicians' perception of teleconsultation modality

**Figure 1:**
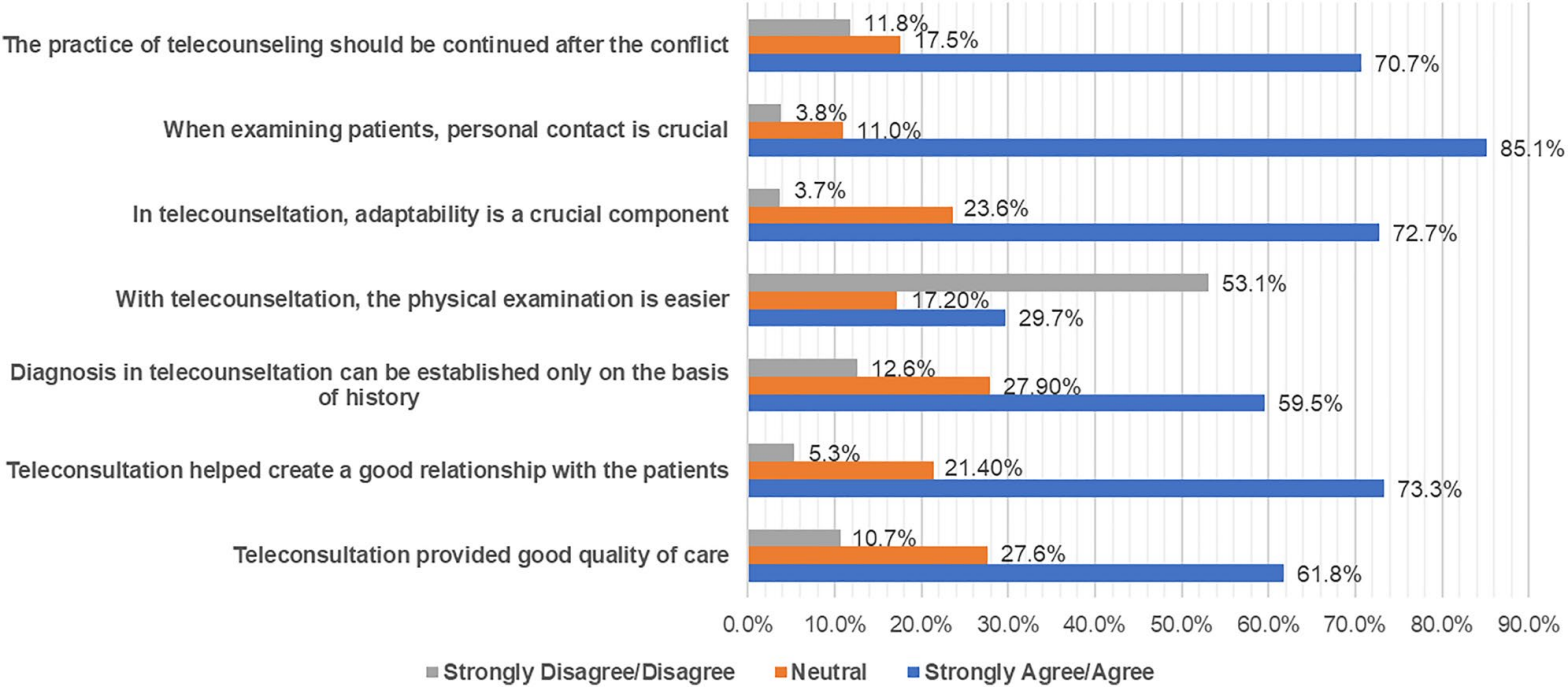
Physician’s perceptions of teleconsultation (N = 2463).

More than half of the participants (61.8%, n = 1521) agreed that teleconsultation provides good quality of care, and 73.3% (n = 1806) agreed it can create trusted patient-physician relations. Moreover, 59.5% (n = 1465) agreed that the diagnosis in teleconsultation could only be made from the patient history, 53.1% (n = 1731) disagreed that it is easily suitable to use for physical exams, 85.1% (n = 2097) agreed that physical touch was necessary in general, and 72.7% (n = 1791) agreed that patients' and doctors' adaptability to these methods was important. Most doctors (70.7%, n = 1742) believed the current teleconsultation experience should continue in Sudan after the war (Fig. 1).

### Physicians’ concerns with teleconsultations

**Figure 2:**
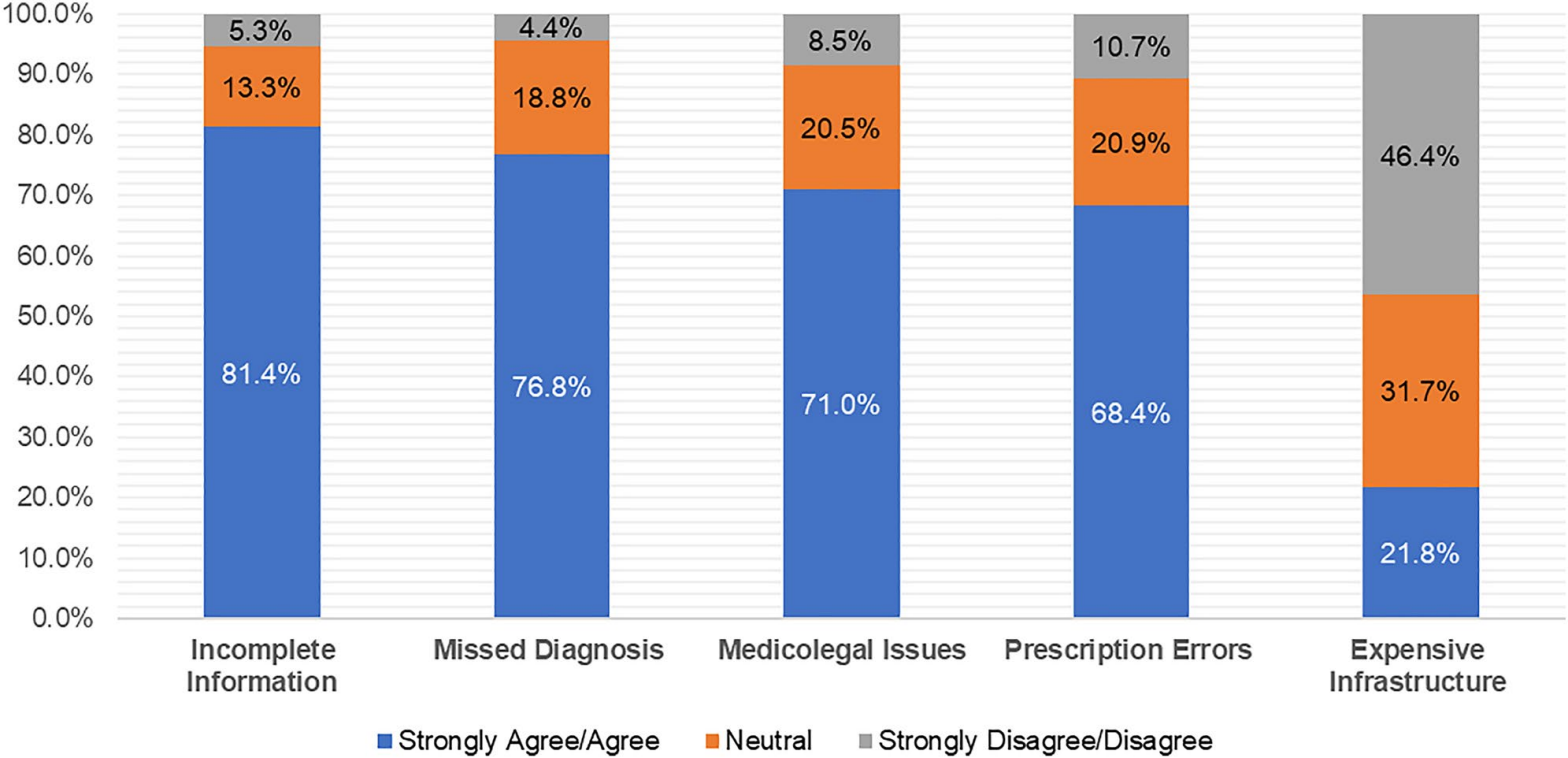
The participant’s concerns over teleconsultation (N = 2463).

The most agreed upon concern over teleconsultation in this study was the fear of incomplete information (81.4%, n = 2005), followed by worries about misdiagnosis (76.8%, n = 1891), medicolegal issues (71.0%, n = 1748), and concerns about prescription errors (68.4%%, n = 1685). The least concerning factor was the cost of teleconsultation infrastructure, with only 21.8% (n = 538) of doctors thinking that it required a high cost of establishment (Fig. 2). As shown in Table 3. The concern scores differed significantly among users of different platforms (p < 0.05). The highest concern scores were among those who used video calls, followed by voice calls, messaging apps, telemedicine software, and emails. On the other hand, no significant difference in the concern score was found in participants with different genders, age groups, years of experience, and departments (p > 0.05).

## Discussion

To our knowledge, this is the first study that provided a comprehensive exploration of the potential and challenges of remote healthcare delivery in resource-restricted and conflict-affected settings. Through an analysis of the technology employed, doctors' perceptions, and their associated concerns, this discussion unveils the intricate dynamics of performing teleconsultation within a crisis context. The implications stemming from these findings contribute to a holistic understanding of the role and prospects of teleconsultation in Sudanese medical practice.

The technology landscape for teleconsultation in the context of the recent conflict in Sudan reveals a variety of platforms and tools being utilized by healthcare practitioners. Voice call was the predominant mode representing half of the teleconsultations, highlighting its accessibility and familiarity among doctors and patients. Messaging applications followed closely at 30.6%, indicating a preference for text-based interactions that can be conducive to asynchronous communication. Video call platforms were especially appreciated for their user-friendly nature, the option to accommodate multiple participants, and seamless integration with essential tools like calendars and emergency services. Furthermore, the capability to share screens emerged as a significant benefit of video and telemedicine software, enhancing the effectiveness of remote consultations. In contrast, messaging applications were favoured for their capacity to engage in direct patient communication, underscoring the value of quick, patient-centered interactions. Moreover, the statistical superiority of video platforms in terms of convenience reinforces their potential to offer a more comprehensive teleconsultation experience. Conversely, messaging applications exhibited a lower convenience score, suggesting potential limitations in fulfilling complex healthcare requirements solely through text-based exchanges. The diversity of preferences among these technologies in our study may reflect a difference in the perceived advantages of each of the platforms, or a perceived ease of usage for both physicians and patients and may also reflect the dynamic nature of teleconsultation during crisis scenarios. This warrants careful consideration when designing future telemedicine interventions. As such, harnessing a combination of these technologies could potentially optimize the balance between accessibility, efficiency, and the quality of patient care.

Our study findings support the conclusions of a recent systematic review that compared face-to-face clinic visits with telephone or video consultations and showed that phone and video consultations were as satisfying and equally efficient as in-person appointments, particularly for primary care medical visits and for persons with mental health disorders^19^. As with our study, two other recent systematic reviews showed that the most typical method of teleconsultations was over digital ISDN lines, followed by smartphone teleconsultation apps^20,21^. The use of mobile apps and messaging received frequent positive regard for teleconsultations for mental health especially^22^. However, a fair comparison between these different methods of teleconsultation must take into account how these different methods of conveying information may promote or discourage clinical work-up, medication-seeking, and further help-seeking behaviour. For typical primary care illnesses treated via telemedicine, a study showed that there are statistically significant differences in clinician orders by teleconsultation method. Additionally, for certain conditions, visual information conveyed during video visits has promoted clinical work-up and treatment in comparison to other methods^23^.

The perception that diagnostic decisions could only be made based on patient history in teleconsultation (59.5%), and teleconsultation may not be as readily suitable for physical examinations with many doctors (85.1%), underscores the general necessity of physical touch in medical practice. This highlights the practitioners' less confidence in extracting valuable clinical information during teleconsultation echoing the challenges of assessing certain aspects of patient health remotely. Encouragingly, a significant majority (70.7%) of medical professionals envision a lasting role for teleconsultation in the post-conflict era in Sudan, implying a recognition of the enduring value and potential enhancements that these remote practices could offer even beyond the immediate crisis period. These collective perceptions offer insights into the balance between the merits of teleconsultation and the enduring importance of physical interaction in healthcare, thus shaping discussions around the sustainable integration of these practices in Sudanese medical systems moving forward.

Because diagnostic decision-making depends on history taking and physical examination, the reliance solely on patient history in diagnosis was reported as problematic by physicians and in our study. The limitations of physical examination in teleconsultations are undeniable and should be taken into consideration and disclosed openly to patients^24^. Other studies on teleconsultations also confirmed that the major concern of physicians was the quality of physical examination^25^ and the diagnosis was accurate only as much as 77% in comparison to in-clinic diagnosis in fields like dermatology^26^ the current study’s findings.

Studies on physicians' perceptions of teleconsultations also included additional aspects, such as major drivers for physicians to engage in teleconsultation with patients, which were time-saving, increased productivity, improvements in patient's health and patient management, health system cost reduction. A study of general practitioners conducted by Segui et al.^27^ reported that the ease of access increased the demands for healthcare support for 27% of cases that patients might have not initiated queries about, and calculated that delivering teleconsultations through an efficient platform could replace 63–88% of conventional appointments^27^. The most frequent uses of teleconsultations were for the management of test results, medical queries, and repeated prescriptions^27^. Studies in paediatric care reveal that teleconsultation has a significant potential for application in guiding differential diagnosis and management, visual diagnosis, management of low-frequency high-stakes occurrences, determining the severity of sickness, and other areas. Having restrictions like the technology's rare use and some implementation roadblocks^28^. In the fields of maternal and neonatal care, a study conducted in Bangladesh found that through teleconsultations general practitioners cleared up myths and encouraged responsible medication use, regular wellness checks, and good healthcare practices. In addition to advising families to seek care at health facilities for urgent or semi-urgent conditions, they assisted families in understanding the severity of illnesses^29^.

On the other hand, the apprehensions surrounding teleconsultation among Sudanese medical practitioners during the conflict period were notably diverse and multifaceted. The most prevalent concern, shared by a substantial 81.4% of participants, pertained to the perceived challenge of obtaining comprehensive patient information remotely. This apprehension underscores the significance of physical examination and the difficulty of capturing nuanced clinical cues solely through virtual interactions. Likewise, the risk of misdiagnosis also engenders substantial worry among a notable 76.8% of respondents. This fear is indicative of the doctors' understanding of the limitations of remote diagnosis, especially when dealing with complex or ambiguous cases. The prominence of concerns related to medicolegal ramifications (71.0%) and prescription errors (68.4%) also suggests a deep-seated awareness of the legal and ethical dimensions associated with teleconsultation. These concerns underline the need for robust frameworks that ensure patient safety, data privacy, and accurate prescription practices in a digital healthcare landscape. Interestingly, the relatively diminished concern regarding the cost of telemedicine infrastructures (21.8%) hints at a perceived feasibility of implementing these technologies, possibly driven by the urgency of the conflict scenario. The variance in concerns among users of different platforms emphasizes the nuanced challenges associated with each mode of communication, underscoring the need for tailored strategies to mitigate these worries. Notably, the lack of significant differences in concern scores based on gender, age, experience, and department suggests a commonality of anxieties that transcends demographic factors. This convergence in concerns highlights the overarching need for comprehensive training, standardized protocols, and meticulous quality assurance measures to address these apprehensions systematically. As teleconsultation continues to evolve and integrate within Sudanese medical practice, these apprehensions provide valuable insights for the refinement of telemedicine strategies, ensuring a balanced approach that leverages the benefits while mitigating potential drawbacks.

As was observed in our study, earlier studies also showed that clinical examination via telemedicine and e-health is particularly difficult, and may sometimes be unfeasible altogether^30^. However, this current limitation may 1 day be handled by the use of emergent innovations and telemedicine peripherals which are currently only available in high-income settings, but would likely remain inaccessible in low-income settings including Sudan^31^. Additionally, clinical evaluations like weight and height measures and taking vital signs are not feasible through teleconsultation, which may be critical in some fields of medicine for diagnosis^32^. Nonverbal cues are also missed in teleconsultations, which encourage hospital-based specialists particularly to lean toward in-person consultation^30^. Other concerns included legal issues, technology costs, sustainability, and the lack of national technology infrastructures which were among the worries and difficulties identified in the literature and also highlighted in our study^13^. As was also the case in our study, other issues often highlighted as key areas of concern for physicians were quality of care, access, consent, adaptability, and privacy^33,34^. Conversely, our physicians' concern about prescription errors was not found in other literature. Pertinently, many of the observed issues and difficulties in teleconsultation may be exacerbated by the absence of key players in the Sudanese teleconsultation program, which are present in other telemedicine models^35^. Despite these challenges, the Sudanese Ministry of Health should assume the important role it can play in grounding these initiatives by planning, providing logistical support, coordinating and mobilizing healthcare professionals as well as funding these initiatives.

### Limitations

While this online survey provided valuable insights into the perceptions and concerns of Sudanese medical practitioners regarding teleconsultation during the conflict, several limitations should be acknowledged. The study’s reliance on online data collection and non-probability sampling procedures may have introduced selection bias, as participation was contingent on internet access and familiarity with online surveys, potentially excluding those without internet access. The self-report nature of the survey further widens the possibility of response bias, where participants may provide socially desirable answers or misrepresent their actual experiences. Further, the study's scope primarily focused on the perceptions of doctors but lacked patients’ perspectives that could have provided a more comprehensive understanding of teleconsultation adoption in times of conflict. As the survey was conducted amidst conflict, participants' responses could have also been influenced by heightened stress and unique contextual factors. Furthermore, the reliance on quantitative data limits the depth of qualitative insights.

### Conclusion

In the face of national conflict, Sudanese physicians view teleconsultation as a viable option to sustain patient care, despite challenges such as the inability to conduct physical examinations and the potential for misdiagnosis. This study affirms teleconsultation's value, while also noting the necessity for proper planning and training to mitigate risks. The study's findings transcend demographic boundaries, emphasizing the need for standardized training and protocols to optimise the implementation of teleconsultation modality as part of routine healthcare delivery in Sudan and in the twenty-first century. It suggests that a well-designed teleconsultation platform can optimize patient care in crisis situations. Despite the limitations of this survey-based study, the findings offer significant insights for integrating teleconsultation into Sudan's healthcare system, with long-term benefits beyond current conflicts. Looking ahead, it is of enduring relevance post-conflict, to assist in paving the way for a dynamic and flexible patient-centred healthcare landscape, bridging technology and tradition to elevate patient outcomes and resilience.

## Methods

### Study design

We conducted a descriptive cross-sectional online survey of Sudanese doctors using convenience sampling. The electronic survey was published on different social media platforms between 18 and 25 July 2023 and could be accessed by anyone with the link. Information about the study was shared with a group of collaborators to facilitate data collection. Initial contact was not made with respondents before commencing the study. Study information inviting individuals to contribute to a study that investigated the experience of providing teleconsultation during the conflict in Sudan was disseminated, including the Participant Information Sheet (PIS) and link to the survey. The PIS included information regarding the study's aims, the protection of participants' personal data, survey length, and their right to withdraw from the study at any time. Participants were informed that this was a voluntary survey without any monetary incentives. The target population included Sudanese medical professionals, encompassing officers, residents, specialists, and consultants holding a practice license from the Sudan Medical Council, irrespective of their location within or outside Sudan, and conducting teleconsultations with Sudanese patients during the conflict period (first 3 months of conflict). Inclusion criteria were only validated in the survey asking participants to participate only if they have provided teleconsultation to Sudanese patients during the conflict period in Sudan.

### Data collection

An online self-administered questionnaire was developed based on recent literature^36–39^, with further input from faculty members of the Department of Public Health and Community Medicine at the University of Bakht Alruda, Sudan. The questionnaire covered various domains, including sociodemographic data, platform usage, clinicians' perceptions, and concerns related to teleconsultations (Supplementary File 1). The questions were distributed with randomization to reduce the possibility of response bias and response validation (completeness check) for all the mandatory items was activated to prevent missed answers in the submitted responses and respondents were able to review and change their answers using a ‘back button’ function. To ensure questionnaire clarity and relevance, a pilot study involving 30 medical practitioners from diverse specialties and academic backgrounds was conducted. Feedback was used to help improve the wording of the initial survey questions although respondents felt the majority of the questions were clear, relevant, and specific.

Data collection was facilitated through Google Forms and distributed by a group of 26 collaborators to personal and professional groups, and via social media platforms including Facebook, WhatsApp, Twitter, and LinkedIn. Study information was also posted on Sudanese social media groups for physicians and reminders to complete the questionnaire were posted on days 3 and 7 of the data collection period. The membership in these groups is meticulously verified through university certificates and residency documentation for practitioners outside Sudan, ensuring the authenticity of participants. Respondents' IP addresses were not collected to maintain anonymity and confidentiality. However, Google Forms only permitted one submission from the same IP address.

### Data management and statistical analysis

Responses were stored in Google Sheets secured with a password. Only the study team had access to participants' responses. The data was cleaned and analysed using the Statistical Package for Social Sciences (SPSS) software version 26 (https://www.ibm.com/docs/en/spss-statistics/26.0.0). Descriptive statistics, including frequencies and percentages, were utilized to summarise survey responses. Convenience and concern scores for teleconsultation platforms were calculated based on a five-point Likert scale ranging from strongly agree to strongly disagree. The Likert score was coded as follows; 0 for "Strongly Disagree", 1 for "Disagree", 2 for "Neutral", 3 for "Agree", and 4 for "Strongly Agree". When summarizing the results of the Likert scale, Strongly Agree and Agree were later grouped into ‘Agree’. Whereas Strongly Disagree and Disagree were later grouped into ‘Disagree’. The total scores ranged from 0 to 28 for convenience and 0 to 20 for concerns. Higher scores indicated higher levels of convenience or concerns, respectively. The Kruskal–Wallis Test was employed to compare platform usage, convenience, and concern scores. Statistical significance was set at the 0.05 level of alpha error.

### Ethical approval

The study received a favourable opinion from the Research and Ethics Committee, University of Gezira, Sudan, adhering to ethical standards outlined in the 1964 Helsinki Declaration and its later amendments and other approved ethical guidelines. Informed consent was included in the data collection tool and obtained from all participants. The Checklist for Reporting Results of Internet E-Surveys (CHERRIES)^40^ was followed to ensure comprehensive and accurate reporting of the study findings, as detailed in Supplementary File 2. We confirm that all methods were carried out according to relevant research ethics guidelines and regulations. Before filling out the questionnaire, all the participants provided informed consent that was included at the beginning of the online questionnaire.

### Supplementary Information

Supplementary Information 1.

Supplementary Information 2.

## Data Availability

The data set used and/or analysed during the study are available from the corresponding author on reasonable request.
