# Supplementary material for: Physician experiences with teleconsultations amidst conflict in Sudan
Source: Sci Rep. 2023 Dec 20;13:22688. doi: 10.1038/s41598-023-49967-5 (PMC10730898; doi:10.1038/s41598-023-49967-5)
Supplement: Supplementary file 1 — Supplementary Information 1. [file 41598_2023_49967_MOESM1_ESM.pdf]

Teleconsultation in Times of Conflict: Experiences, Perceptions, and Concerns of Sudanese Doctors

We are excited to invite you to participate in our research study. This cross-sectional survey is being conducted by the Sudanese Researchers Collaboration Initiative (SRCI), with the aim of understanding the current landscape of tele-consultation adoption among Sudanese medical officers, residents, specialists, and consultants, both within and outside Sudan, who hold a valid practice license from the Sudan Medical Council and provided teleconsultation to patients in Sudan in the past three months.

Please be assured that all information provided will be kept strictly confidential.

Your responses will be anonymized and aggregated for analysis purposes only.

Your identity will not be disclosed in any publication or report resulting from this study.

Your participation is voluntary, and you have the right to withdraw from the study at any point in time.

If you have any questions or require further assistance, please contact the principal investigators: Mohammed Mahmoud (m.mahmmoud96@gmail.com) and Yasir Elhadi (Yasirelhadi25@gmail.com)

* Indicates required question

1. I consent to participate in this study *

Mark only one oval.

- Yes
- No

2. Age (Years) *

Mark only one oval.

- ≤30
- 31- 40
- 41-50
- 51- 60
- >60

3. Sex *

Mark only one oval.

- Male
- Female

4. Level of practice *

Mark only one oval.

- Medical officer
- Registrar
- Specialist
- Counseltant

5. Years in medical practice *

Mark only one oval.

- ≤ 5
- 6- 10
- 11-20
- 21- 30
- >30

6. The most frequent patient population in the past 3 months *

Mark only one oval.

- Adults
- Pediatrics
- Both

7. Provider location *

Mark only one oval.

- Khartoum state
- Other Sudan States
- Africa
- Asia
- Europe
- Northern and Southern America

8. Department *

Mark only one oval.

- Internal medicine
- Family medicine
- Surgery
- Obstetrics and gynecology
- Pediatrics
- Radiology
- Pulmonary
- Intensive care
- Orthopedic
- ENT
- Dermatology
- Emergency Medicine
- Oncology
- Dentistry
- Psychiatry

Platform Usage Data

Please answer the following questions regarding the most frequent platform used

9. Most frequent platform used *

Mark only one oval.

- Phone Call
- Video
- Messages apps
- Telemedicine software
- Email

10. Ease of use *

Mark only one oval.

- Strongly agree
- Agree
- Neutral
- Disagree
- Strongly disagree

11. Ability to have multiple people on the same call *

Mark only one oval.

- Strongly agree
- Agree
- Neutral
- Disagree
- Strongly disagree

12. Ability to share screen *

Mark only one oval.

- Strongly agree
- Agree
- Neutral
- Disagree
- Strongly disagree

13. Good customer service *

Mark only one oval.

- Strongly agree
- Agree
- Neutral
- Disagree
- Strongly disagree

14. Ability to message patients *

Mark only one oval.

- Strongly agree
- Agree
- Neutral
- Disagree
- Strongly disagree

15. Integration with calender *

Mark only one oval.

- Strongly agree
- Agree
- Neutral
- Disagree
- Strongly disagree

16. Integration with emergency *

Mark only one oval.

- Strongly agree
- Agree
- Neutral
- Disagree
- Strongly disagree

Clinician's Perception of Teleconsultation

Please indicate your responses to the following questions regarding teleconsultations done in the past three months in Sudan

17. Teleconsultation provided good quality of care *

Mark only one oval.

- Strongly agree
- Agree
- Neutral
- Disagree
- Strongly disagree

18. Teleconsultation helped create a good relationship with the patients *

Mark only one oval.

- Strongly agree
- Agree
- Neutral
- Disagree
- Strongly disagree

19. Diagnosis in telecounseltation can be established only on the basis of history *

Mark only one oval.

- Strongly agree
- Agree
- Neutral
- Disagree
- Strongly Disagree

20. With telecounseltation, the physical examination is easier *

Mark only one oval.

- Strongly agree
- Agree
- Neutral
- Disagree
- Strongly disagree

21. In telecounseltation, adaptability is a crucial component. *

Mark only one oval.

- Strongly agree
- Agree
- Neutral
- Disagree
- Strongly disagree

22. When examining patients, personal contact is crucial *

Mark only one oval.

- Strongly agree
- Agree
- Neutral
- Disagree
- Strongly Disagree

23. The practice of telecounseling should be continued after the conflict *

Mark only one oval.

- Strongly agree
- Agree
- Neutral
- Disagree
- Strongly disagree

24. Concerns about missed diagnoses arise with telecounseltation. *

Mark only one oval.

- Strongly agree
- Agree
- Neutral
- Disagree
- Strongly Disagree

25. There are worries regarding incomplete information when using telecounseltation. *

Mark only one oval.

- Strongly agree
- Agree
- Neutral
- Disagree
- Strongly disagree

26. Concerns regarding prescription errors arise with telecounseltation *

Mark only one oval.

- Strongly agree
- Agree
- Neutral
- Disagree
- Strongly disagree

27. Medicolegal issues arise with telecounseltation. *

Mark only one oval.

- Strongly agree
- Agree
- Neutral
- Disagree
- Strongly disagree

28. The infrastructure associated with teleconsultations is expensive *

Mark only one oval.

- Strongly disagree
- Disagree
- Neutral
- Agree
- Strongly agree

This content is neither created nor endorsed by Google.

Google Forms
